# Ethical Issues of Risk Management Disclosure in Healthcare Networks

**DOI:** 10.1177/08404704251404875

**Published:** 2026-01-31

**Authors:** Laurie Bouchard, Béatrice Godard

**Affiliations:** 1 5622 School of Public Health, University of Montreal, Montreal, Quebec, Canada

## Abstract

Risk management disclosure is one of the so-called ethical processes that illustrate the culture of fairness (just culture) and no-blame. On the field, however, this culture is not always felt by staff. Disclosure is open to criticism and difficulties and raises ethical issues such as fears of being blamed and fears of the consequences for users. These criticisms and difficulties are linked to ethical values and principles, as is disclosure itself. Thus, many ethical dilemmas are experienced by staff. Fortunately, it is possible to optimize the disclosure process by examining the possibilities offered by organizational ethics to optimize the disclosure process. Using the concepts and tools of organizational ethics helps to overcome the limitations of the risk management process as a whole and to optimize it. It is therefore reasonable to hypothesize that organizational ethics can help to do the same for disclosure.

## Introduction

Beneficence, dignity, users’ rights, caregivers’ rights and duties, fairness, honesty, justice, respect, security and transparency are just a few examples of the values and principles that have been put forward in Quebec’s health and social services system, and which must form part of the system’s organizational culture. Risk management was initiated by the Ministry of Health and Social Services (hereinafter “MHSS”) to ensure the quality and safety of care and services in health and social service establishments (hereinafter “establishments”). It is defined as an organizational process designed to reduce the risk of undesirable events for users. The term “user” refers to a person receiving care or services in a health and social services establishment. An undesirable event is one that should not have occurred, and which may have consequences for users.^1-3^ Here are a few examples: falls, medication errors, documents not transmitted, and confidentiality not respected. In 2002, Quebec amended its Act Respecting Healthcare Services and Social Services (hereafter “ARHSS”) to include risk management.^1-3^

Risk management is about creating and maintaining a fair, non-blame organizational culture. A just culture is the hallmark of an organization that recognizes the difference between an act committed in good faith and a wrongful act. A no-blame culture will focus primarily on systems, not staff.^4,5^ The term “staff” refers to all employees, managers, physicians, professionals, and others who are employed by or under contract with an establishment. As such, MHSS and its establishments are putting in place policies, procedures, and processes that exemplify this just and non-blame organizational culture. Disclosure within risk management is one such process.

Why disclosure in particular? In a few words, disclosure consists of informing users or legal representatives of the nature of an adverse event that occurred during the provision of care or services.^4-7^ It is mandatory under the ARHSS (now called “Health and Social Services System Governance Act”), but also under other legislation and accreditation criteria. It is linked to several values and ethical principles. It is also an institutional and professional responsibility. It is part of a process demonstrating that facilities and practitioners are accountable for their actions, and that suboptimal aspects will be corrected.^4-7^

Despite the willingness of facilities to implement such an organizational culture, the experience of several staff demonstrates a culture where they feel that the principles and values mentioned above are not respected. This can be seen in the disclosure process, which is the subject of much criticism and presents several difficulties. These criticisms and difficulties are various fears of staff, especially in terms of blame and consequences for users.^8-18^ As a result, these varied fears demonstrate that the just and non-blame culture that facilities advocate is not always present, or at least, staff do not always feel that it is, despite the evidence that facilities bring to the MHSS or during accreditations.

First, we’ll propose a definition of organizational culture in health and social services, then we’ll clarify what a just and non-blame culture is, and its place in organizational culture. Next, we define risk management disclosure, its importance in the risk management process and its components, followed by related compliance and legal articles. Finally, we’ll look at the ethical principles and values associated with disclosure, as well as criticisms levelled at disclosure. Finally, we propose some implications for leadership practice. We conclude with the possibility that organizational ethics could be one of the approaches to consider optimizing disclosure within establishments.

## Methodological Approach

The literature and other sources were selected based on the links between the concepts illustrated in the article and were identified using the PubMed, Cochrane, and Medline bibliographic databases. Given the limited literature on these concepts, the selection of references was extended over a period of 10 years. Searches were also conducted in government publications. Some of the references used in this article are older but remain important because they are still in use today.

## A Just and Non-Blaming Organizational Culture and Its Organizational Concerns

Organizational culture can be described as the set of beliefs, values, and attitudes of a company, which are shared by all staff and influence their behaviour.^19^ This culture is passed on to new staff, via training, and reminded to staff during ongoing training or team meetings. It indicates how caregivers should act or react.^19^ An important aspect of an establishment’s organizational culture is the notion of a just and no-blame culture, based on the establishment’s missions and values, as well as the duties of staff and breaches of these duties.^7^

Differences are established between intentional actions, recklessness, and unforeseen circumstances in care complications.^7^ The development of a just and no-blame culture is more effective in ensuring good risk management, since staff are treated fairly and held accountable for their actions and behaviours towards users, according to their role. They are not punished for mistakes or errors, unless they have been negligent or have intentionally acted in an unprofessional manner. A learning system is in place to understand why mistakes happen so that they can be corrected and learned from.^7^

**Figure 1.**
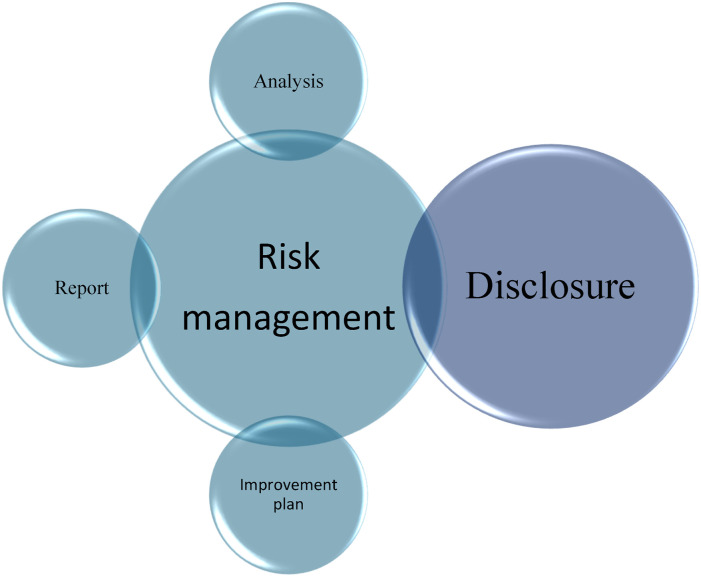
Risk Management and its Different Aspects

A just, no-blame culture will focus its attention on policies, procedures or systems, not on staff.^7,20^ It encourages staff to make their voices heard, and managers to make everyone’s voice heard, as well as fostering constructive discussion and increasing accountability, thus reducing undesirable events.^7,20-22^ It supports a system of shared responsibility in which establishments are accountable for the systems they design and can respond to the behaviours of their staff in a fair and equitable manner. This responsibility is shared between different stakeholders—managers no matter what their level is in the establishment, staff—to the extent that they have the capacity to act.^7,20,21^ If recommendations are to be made to improve service quality and patient safety, they will be made at both individual (e.g., training for staff or managers) and organizational levels (e.g., review a policy or a process, such as fall prevention), and in a bidirectional way (from the field to senior management, and from senior management to the field).^7,21^ An example of a just and no-blame culture is disclosure, an important aspect of the risk management process, as illustrated in Figure 1.

## Organizational Concerns

Organizational concerns relate to the values promoted by the establishments. Despite the fairness and no-blame culture advocated by the establishments, staff are worried about not being approved by their colleagues or managers, being isolated and experiencing harassment.^8-18^ Added to this are fears of being blamed, of having complaints made against them by their colleagues or managers, but also by users and relatives. They fear for their own reputation and that of the establishment. As a result, they experience a culture of blame rather than a culture of fairness and non-blame.^8-18,22^ Given that a court of law may use the words or writings of hospital staff following an adverse event, this encourages defensive communication to protect themselves from external stakeholders. The literature on adverse events reports very little on how hospitals deal with this contradiction,^12^ and focuses mainly on physicians, whereas many other staff are called upon to make disclosures.^12^

## Disclosure: Part of the Risk Management Process, Definition, and Components

We are referring here to risk management communication with users and relatives, and more specifically to disclosure. Disclosure is the standard institutional process of informing users or their legal representatives of the nature of the adverse event, and the means of avoiding its recurrence.^4,6,23^ Disclosure is mandatory for accidents with severe, temporary, or irreversible consequences, such as fall with head injury or fracture, for example.^4,6^ However, best practice recommends disclosure in the case of accidents with no consequences, in order to optimize user safety.^4,10,15^ Every establishment should have a policy and procedure dedicated to disclosure.^23^ In addition to disclosure, risk management includes other communication mechanisms, such as reporting, analysis, and improvement plans. This article focuses solely on disclosure. For the purposes of this article, the term refers only to the risk management process.

Disclosure involves open discussion of an adverse event between users, relatives, and staff in the establishment concerned.^4,23^ It should be seen as an ongoing dialogue and communication process.^4,23^ Disclosure is a process requiring an interdisciplinary dimension. Indeed, professional collaboration must be at the heart of the disclosure process, as each person brings her own work experience, knowledge and skills to the table, forming the richness of a team that can support the users, families, and staff involved, as they have their experiential knowledge of the situation.^4^ Disclosure can help reduce feelings of abandonment and acknowledge suffering and grief, as stakeholders feel they are taken into consideration.^24^

The fact that disclosure takes place in so many establishments and in many countries, such as the United States of America,^10,15^ South Korea,^17^ United Arab Emirates,^12^ Kuwait,^20^ and Canada,^25^ with mostly the same processes testifies to the importance of professionalism and honesty in communicating with users and relatives. This has the effect of amplifying the opportunities for partnerships with the latter.^6,10,23^ Establishing a partnership with users and relatives is important both to prevent a similar event from happening again, or at least to mitigate the risk, and to respect users’ dignity and autonomy.^23,24,26-28^ Apologies and explanations in a vocabulary understood by all are crucial to avoid consequences worse than the adverse event itself, such as anxiety, guilt, or even post-traumatic stress disorder.^4,10-12^ For users and relatives, safety principles are even more important when care and services do not go as planned.^4,27-30^

## Disclosure: Related Compliance and Legal Articles

In Canada, disclosure is a quality criterion of Accreditation.^31^ Accreditation Canada is a healthcare and services accreditation organization. Accreditation helps healthcare establishments understand how to make better use of their resources, increase efficiency, improve quality and reduce risk. It is also an obligation under several ethical and legal texts. Article 8 of the Act respecting health services and social services in the province of Quebec states that users have the right to be informed of any event that has occurred, of the consequences that this event has brought and may bring, of the measures taken to counter these consequences or, at least, to mitigate them, and of the measures taken to avoid recurrence.^32^ The right to information is an integral part of user rights. Article 235.1 stipulates that the board of directors of an establishment must provide for a bylaw on disclosure, which includes the necessary information to be given to users and legal representatives, support measures and measures put in place to prevent recurrence. In the Act respecting the governance of the health and social services system, sections 91, 346, and 1,489 stipulate that disclosure is an obligation for establishments, which must adopt regulations to this effect.^33^ Finally, article 10 of the Civil Code of Quebec states that every person has the right to inviolability and integrity, and that a person cannot be harmed without his or her free and informed consent.^34^

Codes of ethics for health and social service professionals also require disclosure.^2^ *The Canadian Medical Association’s Code of Ethics* stipulates that physicians must disclose to users if they are the victim of an adverse event.^35^ Article 56 of the *Code of Ethics of Quebec Physicians* states that physicians must inform users and their legal representatives of any accident that has had significant (temporary or irreversible) consequences for their health or physical integrity.^36^ Article 12 of the *Code of Ethics for Midwives* stipulates much the same thing^37^ and article 12 of the *Code of Ethics for Nurses* specifies that the event may result from intervention or omission. This article adds that it is the nurse’s duty to document the disclosure in the file, and that she is liable to be penalized for any breach of ethics.^38^

## Disclosure: Associated Ethical Principles and Values, with Criticisms and Difficulties Related to Disclosure Process and Associated with these Ethical Principles and Values

Disclosure is linked to certain ethical principles and values, in addition to reflecting a fair and non-blaming culture in an establishment. Meanwhile, this can also be the subject of some criticisms and difficulties associated with disclosure itself and related to these ethical principles and values. Establishments have policies and procedures surrounding disclosure and promote a culture of fairness and non-blame, as required by accreditation organizations. Nevertheless, there are little data on the number of undisclosed events.^10^

## Value of Autonomy and the Principle of Self-Determination as well as Values of Trust, Honesty, and Transparency

Disclosure is linked to the value of autonomy and the principle of self-determination, which refer to respect for the freedom and right of everyone to choose and act according to his or her own rules.^39-42^

For staff, the value of autonomy and the principle of self-determination imply an obligation to disclose information.^39-42^ Disclosure is linked to the user’s right to information, which is one of the rights enshrined in the ARHSS^32^ and closely associated with the principle of self-determination. Indeed, most users want to know all the facts associated with an adverse event, and if they have suffered harm, they have a right to know and to seek possible redress.^4^

For users, the value of autonomy and the principle of self-determination mean the ability to make choices, validated by free and informed consent and to look after their interests.^41^ Users and relatives are more likely to take legal action when they believe facts have been concealed from them. In fact, up to 44% of lawsuits against facilities are initiated because of a perceived lack of transparency on the part of the pursuers, or because of their desire to know more about the situation.^4,11,12,15,43^

Also, disclosure allows users to become more involved in their care. They feel more like partners than patients. As a result, the quality of care is enhanced.^23,24^ For example, users can be part of the improvement plan and bring their ideas, or they can choose the treatment or support measures they wish after an adverse event. These support measures can be varied, from psychological support to parking payment.

Since disclosure involves presenting users with information that concerns them, it is associated with the values of trust, honesty, and transparency. Since the relationship between staff and users is based on trust, users expect staff to act honestly and respectfully.^23,24^ The response of staff is crucial to users’ recovery.^11^ Conversely, users should not withhold information about their situation, as this could alter the response of caregivers and jeopardize their recovery.^11^ On all sides, non-disclosure leads not only to mistrust, but also to reputational damage.^11,43^

### Organizational Concerns

One of the concerns of an organizational nature is the tension between users’ right to information and duty of honesty and transparency, on the one hand, and, on the other, the desire of establishments to protect their interests.^25^ Staff, too, have a duty to inform, to be honest and transparent, and a desire to protect their interests or prevent emotional consequences that may be difficult for them to bear.^25^ In some cases, it is managers who decide when and what to disclose, and it is they who make the disclosure to users. As a result, the staff involved are not informed of the content of the disclosure, find themselves in a difficult position, and may experience an ethical dilemma between their establishment’s decision and the loyalty they demonstrate towards them and their duty to inform and support the users concerned. In addition, managers disempower staff and deprive them of their key role in consent and disclosure, which risk undermining the relationship of trust between staff and users. Staff may also fear that managers don’t have all the information, or don’t know how to intervene with certain users. There is therefore a tension between the “real culture” of the establishment and the staff duty, who are part and parcel of the organizational values and culture of fairness and non-blame.^7,10^ What’s more, for reasons of protection or convenience, some staff may choose not to disclose,^44^ not to mention the ethical distress they may experience.^44^

Many staff feel that establishments offer insufficient guidance and support when it comes to disclosure.^10^ Guidelines for disclosure support remain scattered, even though they are approved by establishments and accreditations.^10^ In some areas, the definition of an adverse event is unclear, leading to further confusion among staff.^10^

### Concerns Related to the Clinical Condition and the Consequences for Users

By not disclosing, staff feel they are protecting users from information that could have a harmful effect on them. They feel they are doing their duty.^10^ Some users may not want to be informed if they are the victim of an adverse event.^10,45^ What’s more, systems are often based on medical and administrative perspectives, which exclude the voice of users. The latter “undergo” disclosure rather than being actively involved in the analysis and regularly feel unable to put forward their own vision of the situation, or even their proposals for improvement.^46^ What’s more, users and relatives may not understand that an event has occurred and may refuse treatment or continue to display certain behaviours.^46^

## Principles of Beneficence and Non-Maleficence

Disclosure is also associated with the principles of beneficence and non-maleficence, so as not to harm users and to ensure their best interests.^39,47,48^ These are important principles for staff, since the adverse event may have caused harm to users. It is therefore important to find the best balance between the benefits and risks associated with disclosure for users, before proceeding with disclosure.^39,47,48^ In return, users must make their views on the perceived risks and benefits known. A benefit perceived by staff may not be a benefit for users.^39,47,48^ For example, a user who fell can prioritize his quality of life instead of his security: he can choose to go take a walk again because he said it helps him, even if a fall can still happen. As for facilities, not disclosing (or not disclosing properly) can counteract efforts to improve user safety.^4,46^ If an adverse event is neither reported nor disclosed, the necessary follow-up cannot be carried out, and this type of situation could recur.^4,46^

### Concerns Related to the Clinical Condition and the Consequences for Users

Some staff’ fears about disclosure are linked to the clinical condition of users and their families, such as intellectual disability, autism spectrum disorder, or mental health,^13,45^ to certain age groups, such as young people,^16^ or to certain environments, where the user’s condition does not always lend itself to disclosure, such as the emergency department.^22^ In many respects, staff do not see the benefits of disclosure for certain users. They fear creating unexpected reactions, such as anxiety, over-reaction or behavioural problems. Also, if a disclosure is not made properly, with the right people and the right words, it may bring more difficulties, both for user and staff.^46^

## Principles of Justice, Fairness, and Equity

Disclosure also refers to the principles of justice and equity, from the perspective of the common good. This means being impartial when it comes to allocating resources, and applying the rules associated with this allocation. These rules must be fair and the same for everyone.^39,40,47^ Users are entitled to a minimum level of care, and all must be able to receive the care associated with their condition. Staff must respond to needs in an appropriate and timely manner.^39,40,47^ As far as disclosure is concerned, users who are victims of an adverse event are entitled to adequate disclosure, at the earliest possible opportunity (we need to give time to the user, as he must feel ready for disclosure) and by the right people (e.g., for a medication wrong dosage, a physician and a nurse can explain the consequences and the appropriate treatment), especially as this is provided for by law.^49^

### Organizational Concerns

Whatever the situation, it’s important to consider the impact of disclosure on resource allocation: despite themselves, users require more resources and more time, with knock-on effects for other users.^49^

## Ethical Issues

**Figure 2.**
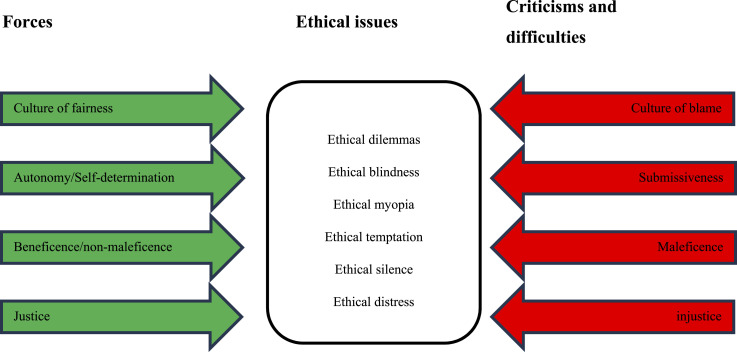
Force-Field Analysis: Disclosure

The forces and the criticisms and difficulties described for disclosure bring ethical issues for staff and managers. Figure 2, based on a tool called “force-field analysis” used in the Lean method to help a team analyze the “forces” at play and arrive at a solution or consensus when faced with a problem to be solved or a proposal for change,^50,51^ helps illustrate the ethical issues that may arise.

There are six main categories^52^:

• Ethical dilemma is defined as a conflict between two or more principles or values.^47,52^ Pursuing one principle or value prevents another principle or value from being realized, which complicates the issue.^47^ Here are two examples: disclosing information when it is required by law and enshrined in an establishment’s values, or not disclosing information, which could protect a user with certain difficulties, as described above, or hesitating to disclose information for fear of blame from the manager or establishment or fear of legal action by the user.
• Ethical blindness is defined as a staff member’s failure to perceive principles or values at stake in a situation or even those that guide their thinking about that situation. In other words, this staff is not sensitive to certain values.^52^ An example here could be an employee who makes a disclosure only out of obligation, without concern for what that disclosure might do to the user.
• Ethical myopia is defined by the fact that a staff member believes that others should share their values, both colleagues and users. This person thus imposes their view of things.^52^ An example here would be to proceed with disclosure using a pre-determined plan, thus presenting the user with an accomplished fact. The user cannot ask questions or make choices. Another example would be a manager forcing an employee to disclose information, ignoring the employee’s concerns about the difficulties that disclosure could cause for the user.
• Ethical temptation is defined as an ethical option vs. a more or less ethical, or even unethical, option.^52^ A staff member is tempted to favour their own interests or those of the establishment at the expense of the user. An example here would be not to disclose at all in order to preserve the reputation of the staff and the institution. Another example would be a manager’s decision to make a partial disclosure, which does not comply with best practices, in order to “meet obligations” while safeguarding the reputation of staff and establishment. The user, who lacks information, cannot make informed choices.
• Ethical silence is defined as a staff member observing an ethical breach but keeping it to themselves.^52^ An example would be a staff member observing that a disclosure has not been made or has been made by colleagues but is incomplete and does not comply with best practices. This staff member decides not to report it to manager.
• Ethical distress is defined as a situation in which a staff member knows what they should do to act appropriately but encounters obstacles that prevent them from doing so.^52^ An example would be an employee who wants to disclose information but is prevented from doing so by manager, who fears the reaction of senior management and for the reputation of the establishment.

## Implications for Leadership Practice

Considering the ethical issues disclosure can cause, not to mention consequences of these difficulties for employees and managers, especially if it is not carried out or if it is not carried out according to best practices. These consequences can include burnout, professional distress, or mistrust of the establishment.^52^

Here are some recommendations:

• Introduce spaces for reflexion and provide training and support for staff, but also for managers^53^: Health and social service establishments have introduced such initiatives about other topics. Why not about disclosure? These space for reflexion and training courses given by risk managers, and can include case studies, analytical models, experiential development, discussions on organizational values, and improving practices.^54^
• Ensure the active involvement of users and their families in the risk management process.^53^ Users’ contribution to risk management enables a more complete analysis of the situation and concrete recommendations that reflect their own experience and perception of the situation.^55^ Some users may even mention aspects of analysis that professionals had not considered.^53^

## Conclusion: Perspectives Offered by Organizational Ethics

A culture of fairness and no-blame is an integral part of an ethical culture, and one that establishments are keen to put into practice. Disclosure, an integral part of risk management and both ethical and deontological obligations, reflects this culture of fairness and no-blame. However, this culture is not always felt in the field, and this is reflected in disclosure, which can lead to several criticisms, difficulties and ethical issues. Ethical values and principles, and the ethical issues raised by disclosure, illustrate that many of these are organizational in nature. One approach to alleviating these fears, difficulties, and issues is to draw on the resources of organizational ethics.

Organizational ethics is based on two concepts: ethics and organization.^56^ Organizational ethics involves reflecting on the choice of values to guide management decisions that influence care and services to users, as well as their evolution in a changing environment and clinical practice.^56,57^ Organizational ethics refers primarily to issues of administration, management, compliance, governance, and shared values within an establishment.^58-60^ It aims to influence organizational decisions by adding a form of ethicality.^61^ These decisions have repercussions on users, staff, and the community to which the establishment belongs. Organizational ethics is therefore the articulation, application, and evaluation of the implementation of an establishment’s values and moral positions,^57,62^ which are mentioned in the establishment’s documents, such as mission statements, the managerial code of ethics, or a list of organizational values and their definitions.

Organizational ethics focuses on ethical issues faced by managers and board members of an establishment, as well as the implications of decisions for users, staff, and the community.^58-60,63^ In contrast, clinical ethics and biomedical ethics are more concerned with individual issues, such as ethical questions and conflicts of values between individuals, such as a physician (or other healthcare worker) and the user, or the user and a family member,^58^ while bioethics (not to be confused with biomedical ethics) aims to clarify or resolve ethical questions raised by scientific advances and technological developments not only in the health and social services sector, but in human life in general.^47^

It has been reported that using the concepts and tools of organizational ethics helps to overcome the limitations of the risk management process as a whole and to optimize it.^53,64^ Indeed, the combination of organizational ethics and risk management broadens the scope of the latter.^55,64^ It is therefore reasonable to hypothesize that organizational ethics can help to do the same for disclosure. An empirical research will be held soon to answer that hypothesis.

## Notes and Limitations

This article deals with organizational culture, risk management disclosure, and organizational ethics in the health and social services sector. Integrated risk management, which is still under development in the health and social services sector, is not addressed. Here, risk management, and more specifically disclosure, is focused on patient safety, and the same processes are used in the various healthcare and social services sectors. Furthermore, in the province of Quebec, and particularly in the health and social services network, the field of organizational ethics is still developing and has not yet been sectorized. The link between risk management (disclosure) and organizational ethics is therefore still in its early stages. Also, this article focuses on theoretical concepts and the possible links between them. It is a part of an empirical study which will be conducted shortly to illustrate these concepts and the links between them and recommendations towards it.
